# (*E*)-3-[4-(Dec­yloxy)phen­yl]-1-(4-hydroxy­phen­yl)prop-2-en-1-one

**DOI:** 10.1107/S160053680901441X

**Published:** 2009-04-22

**Authors:** Ibrahim Abdul Razak, Hoong-Kun Fun, Zainab Ngaini, Norashikin Irdawaty Abd Rahman, Hasnain Hussain

**Affiliations:** a X-ray Crystallography Unit, School of Physics, Universiti Sains Malaysia, 11800 USM, Penang, Malaysia; b Department of Chemistry, Faculty of Resource Science and Technology, Universiti Malaysia Sarawak, 94300 Kota Samarahan, Sarawak, Malaysia; c Department of Molecular Biology, Faculty of Resource Science and Technology, Universiti Malaysia Sarawak, 94300 Kota Samarahan, Sarawak, Malaysia

## Abstract

In the title compound, C_25_H_32_O_3_, the enone group adopts an *s*–*cis* conformation. The alk­oxy unit is nearly planar and is in a *trans* conformation. The two benzene rings make a dihedral angle of 18.87 (9)°. In the crystal structure, mol­ecules are linked into chains running along the *a* axis by inter­molecular O—H⋯O hydrogen bonds involving the hydr­oxy and keto groups. The chains are crosslinked along the *b* axis *via* C—H⋯O hydrogen bonds, forming a two-dimensional network parallel to the *ab* plane.

## Related literature

For the biological properties of chalcone derivatives, see: Bhat *et al.* (2005 ▶); Xue *et al.* (2004 ▶); Satyanarayana *et al.* (2004 ▶); Lee *et al.* (2006 ▶). For related structures, see: Ng *et al.* (2006 ▶); Razak *et al.* (2009 ▶); Ngaini, Fadzillah *et al.* (2009 ▶); Ngaini, Rahman *et al.* (2009 ▶). For bond-length data, see: Allen *et al.* (1987 ▶). For the stability of the temperature controller used for the data collection, see: Cosier & Glazer (1986 ▶).
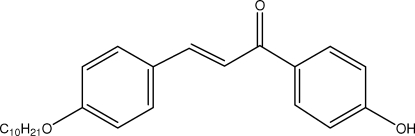


## Experimental

### 

#### Crystal data

C_25_H_32_O_3_
*M* *_r_* = 380.51
Orthorhombic, 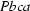

*a* = 10.5192 (3) Å
*b* = 9.9839 (3) Å
*c* = 40.8415 (12) Å
*V* = 4289.3 (2) Å^3^
*Z* = 8
Mo *K*α radiation
μ = 0.08 mm^−1^
*T* = 100 K
0.58 × 0.49 × 0.03 mm

#### Data collection

Bruker APEXII CCD area-detector diffractometer
Absorption correction: multi-scan (*SADABS*; Bruker, 2005 ▶) *T*_min_ = 0.957, *T*_max_ = 0.998
42832 measured reflections
4922 independent reflections
3526 reflections with *I* > 2σ(*I*)
*R*_int_ = 0.082

#### Refinement

*R*[*F* ^2^ > 2σ(*F* ^2^)] = 0.064
*wR*(*F* ^2^) = 0.132
*S* = 1.10
4922 reflections
258 parameters
H atoms treated by a mixture of independent and constrained refinement
Δρ_max_ = 0.20 e Å^−3^
Δρ_min_ = −0.22 e Å^−3^

### 

Data collection: *APEX2* (Bruker, 2005 ▶); cell refinement: *SAINT* (Bruker, 2005 ▶); data reduction: *SAINT*; program(s) used to solve structure: *SHELXTL* (Sheldrick, 2008 ▶); program(s) used to refine structure: *SHELXTL*; molecular graphics: *SHELXTL*; software used to prepare material for publication: *SHELXTL* and *PLATON* (Spek, 2009 ▶).

## Supplementary Material

Crystal structure: contains datablocks global, I. DOI: 10.1107/S160053680901441X/ci2782sup1.cif

Structure factors: contains datablocks I. DOI: 10.1107/S160053680901441X/ci2782Isup2.hkl

Additional supplementary materials:  crystallographic information; 3D view; checkCIF report

## Figures and Tables

**Table 1:** Hydrogen-bond geometry (Å, °)

*D*—H⋯*A*	*D*—H	H⋯*A*	*D*⋯*A*	*D*—H⋯*A*
O1—H1O1⋯O2^i^	0.95 (3)	1.71 (3)	2.655 (2)	177 (3)
C5—H5⋯O1^ii^	0.93	2.48	3.340 (2)	155

Symmetry codes: (i) 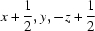; (ii) 
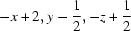
.

**Fig. 1.**
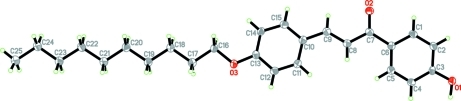
The molecular structure of the title compound, showing 50% probability displacement ellipsoids and the atom-numbering scheme.

**Fig. 2.**
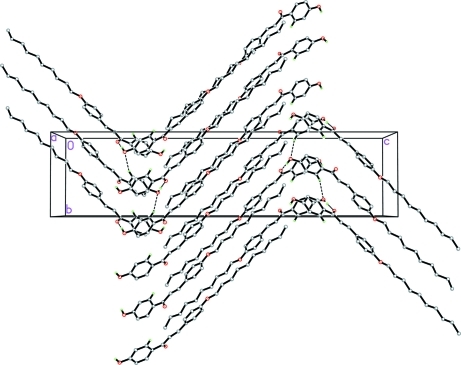
The crystal packing of the title compound, viewed down the a axis. Dashed lines indicate hydrogen bonds.

## supplementary crystallographic information

### Comment

Chalcone derivatives possess a wide range of biological properties such as antimalarial (Xue *et al.*, 2004), antiangiogenic and antitumour (Lee *et al.*, 2006), anticancer (Bhat *et al.*, 2005) and antihyperglycemic (Satyanarayana *et al.*, 2004) activities. Chalcones have been widely studied and developed as one of the pharmaceutically important molecules. We have synthesized the title chalcone derivative and tested and confirmed its activities against *E. coli* ATCC 8739. As part of our studies on chalcone derivatives, we report here the crystal structure of the title compound.

In the title molecule (Fig 1), bond lengths show normal values (Allen *et al.*, 1987). The mean plane through the enone moiety (O2/C7/C8/C9) form dihedral angles of 19.26 (12)° and 2.14 (12)°, respectively, with the C1-C6 and C10-C15 benzene rings. The dihedral angle between the two benzene rings is 18.87 (9)°. The conformation of the enone moiety is *s*-*cis* with O2—C7—C8—C9 torsion angle being 5.4 (3)°. Slight enlargement of C5—C6—C7 (122.43 (18)°) angle is as a result of the short H5···H8 (2.18 Å) contact whereas short H8···H11 (2.30 Å) contact widened the C8—C9—C10 (128.13 (19)°) and C9—C10—C11 (123.63 (18)°) angles. Similarly, strain induced by close interatomic contact between H14 and H16A (2.27 Å) resulted in the opening of O3—C13—C14 (124.86 (17)°) angle. Related structures by Ng *et al.*(2006), Razak *et al.* (2009), Ngaini, Fadzillah *et al.* (2009) and Ngaini, Rahman *et al.* (2009) have also reported similar features.

The zigzag alkoxyl tail adopts a *trans* conformation with the largest deviation from the ideal value being 175.44 (17)° for C17—C18—C19—C20 torsion angle. The alkoxyl chain (O3/C16-C25) is nearly planar with the maximum deviation from the least-squares plane being 0.116 (1) Å for atom C19. The dihedral angle between the O3/C16-C25 and C10-C15 planes is 6.29 (13)°.

In the crystal structure, the molecules are arranged in alternating head-to-head zigzag layers along the *c* axis (Fig. 2). Intermolecular O1—H1O1···O2(*x* + 1/2,*y*,-*z* + 1/2) hydrogen bonds (Table 1) between hydroxy and keto groups form extended chains along the *a* axis. These chains are interconnected along the *b* axis by C5—H5···O1(-*x* + 2,*y* - 1/2,-*z* + 1/2) intermolecular interactions forming a two-dimensional network parallel to the *ab* plane.

### Experimental

A mixture of 4-hydroxyacetophenone (2.72 g, 20 mmol) and 4-decyloxybenzaldehyde (5.25 ml, 20 mmol) and KOH (4.04 g, 72 mmol) in methanol (60 ml) was heated at reflux for 24 h. The reaction mixture was cooled to room temperature and acidified with cold diluted HCl (2 N). The resulting precipitate was filtered, washed and dried. After redissolving in a hexane-ethanol (7:1) solution, followed by few days of slow evaporation, crystals were collected.

### Refinement

The O-bound H atom was located in a difference Fourier map and refined freely. C-bound H atoms were positioned geometrically and refined using a riding model with C-H = 0.93–0.97 Å. The *U*_iso_(H) values were constrained to be 1.5U_eq_(C_methyl_) and 1.2U_eq_(C). A rotating group model was used for the methyl group.

### Crystal data

**Table:**

C_25_H_32_O_3_	*F*(000) = 1648
*M**_r_* = 380.51	*D*_x_ = 1.178 Mg m^−^^3^
Orthorhombic, *P**b**c**a*	Mo *K*α radiation, λ = 0.71073 Å
Hall symbol: -P 2ac 2ab	Cell parameters from 4176 reflections
*a* = 10.5192 (3) Å	θ = 2.2–23.9°
*b* = 9.9839 (3) Å	µ = 0.08 mm^−^^1^
*c* = 40.8415 (12) Å	*T* = 100 K
*V* = 4289.3 (2) Å^3^	Plate, colourless
*Z* = 8	0.58 × 0.49 × 0.03 mm

### Data collection

**Table:**

Bruker APEXII CCD area-detector diffractometer	4922 independent reflections
Radiation source: sealed tube	3526 reflections with *I* > 2σ(*I*)
graphite	*R*_int_ = 0.082
π and ω scans	θ_max_ = 27.5°, θ_min_ = 1.0°
Absorption correction: multi-scan (*SADABS*; Bruker, 2005)	*h* = −13→13
*T*_min_ = 0.957, *T*_max_ = 0.998	*k* = −12→12
42832 measured reflections	*l* = −53→52

### Refinement

**Table:**

Refinement on *F*^2^	Primary atom site location: structure-invariant direct methods
Least-squares matrix: full	Secondary atom site location: difference Fourier map
*R*[*F*^2^ > 2σ(*F*^2^)] = 0.064	Hydrogen site location: inferred from neighbouring sites
*wR*(*F*^2^) = 0.132	H atoms treated by a mixture of independent and constrained refinement
*S* = 1.10	*w* = 1/[σ^2^(*F*_o_^2^) + (0.0368*P*)^2^ + 2.6345*P*] where *P* = (*F*_o_^2^ + 2*F*_c_^2^)/3
4922 reflections	(Δ/σ)_max_ = 0.001
258 parameters	Δρ_max_ = 0.20 e Å^−^^3^
0 restraints	Δρ_min_ = −0.22 e Å^−^^3^

### Special details

**Table:**

Experimental. The crystal was placed in the cold stream of an Oxford Cryosystems Cobra open-flow nitrogen cryostat (Cosier & Glazer, 1986) operating at 100.0 (1) K.
Geometry. All e.s.d.'s (except the e.s.d. in the dihedral angle between two l.s. planes) are estimated using the full covariance matrix. The cell e.s.d.'s are taken into account individually in the estimation of e.s.d.'s in distances, angles and torsion angles; correlations between e.s.d.'s in cell parameters are only used when they are defined by crystal symmetry. An approximate (isotropic) treatment of cell e.s.d.'s is used for estimating e.s.d.'s involving l.s. planes.
Refinement. Refinement of *F*^2^ against ALL reflections. The weighted *R*-factor *wR* and goodness of fit *S* are based on *F*^2^, conventional *R*-factors *R* are based on *F*, with *F* set to zero for negative *F*^2^. The threshold expression of *F*^2^ > σ(*F*^2^) is used only for calculating *R*-factors(gt) *etc*. and is not relevant to the choice of reflections for refinement. *R*-factors based on *F*^2^ are statistically about twice as large as those based on *F*, and *R*- factors based on ALL data will be even larger.

### Fractional atomic coordinates and isotropic or equivalent isotropic displacement parameters (Å^2^)

**Table:**

	*x*	*y*	*z*	*U*_iso_*/*U*_eq_	
O1	0.97999 (14)	0.70636 (15)	0.20018 (3)	0.0232 (3)	
O2	0.66825 (13)	0.56794 (14)	0.32684 (3)	0.0228 (3)	
O3	0.93786 (13)	−0.03516 (14)	0.45190 (3)	0.0227 (3)	
C1	0.79176 (18)	0.6934 (2)	0.27426 (5)	0.0213 (4)	
H1	0.7242	0.7397	0.2836	0.026*	
C2	0.83983 (19)	0.7353 (2)	0.24449 (5)	0.0220 (4)	
H2	0.8051	0.8096	0.2340	0.026*	
C3	0.94058 (19)	0.6659 (2)	0.23015 (5)	0.0198 (4)	
C4	0.9958 (2)	0.5589 (2)	0.24676 (5)	0.0224 (4)	
H4	1.0655	0.5149	0.2378	0.027*	
C5	0.94723 (18)	0.5179 (2)	0.27665 (5)	0.0219 (4)	
H5	0.9847	0.4463	0.2875	0.026*	
C6	0.84262 (18)	0.5826 (2)	0.29070 (4)	0.0193 (4)	
C7	0.77760 (18)	0.5307 (2)	0.32020 (4)	0.0189 (4)	
C8	0.84031 (19)	0.4306 (2)	0.34137 (4)	0.0206 (4)	
H8	0.9248	0.4078	0.3377	0.025*	
C9	0.77621 (19)	0.3725 (2)	0.36590 (4)	0.0204 (4)	
H9	0.6932	0.4018	0.3690	0.024*	
C10	0.82043 (18)	0.2687 (2)	0.38841 (4)	0.0204 (4)	
C11	0.94097 (18)	0.2090 (2)	0.38649 (5)	0.0216 (4)	
H11	0.9981	0.2378	0.3706	0.026*	
C12	0.97590 (19)	0.1086 (2)	0.40773 (5)	0.0222 (5)	
H12	1.0560	0.0698	0.4059	0.027*	
C13	0.89207 (19)	0.0645 (2)	0.43198 (4)	0.0201 (4)	
C14	0.77209 (18)	0.1205 (2)	0.43435 (5)	0.0213 (4)	
H14	0.7153	0.0914	0.4503	0.026*	
C15	0.73770 (19)	0.2212 (2)	0.41247 (4)	0.0223 (4)	
H15	0.6567	0.2581	0.4139	0.027*	
C16	0.85615 (19)	−0.0838 (2)	0.47766 (5)	0.0223 (5)	
H16A	0.7759	−0.1141	0.4686	0.027*	
H16B	0.8391	−0.0130	0.4933	0.027*	
C17	0.92412 (19)	−0.1985 (2)	0.49413 (5)	0.0226 (5)	
H17A	1.0055	−0.1674	0.5023	0.027*	
H17B	0.9403	−0.2684	0.4782	0.027*	
C18	0.84727 (19)	−0.2561 (2)	0.52231 (5)	0.0241 (5)	
H18A	0.7647	−0.2837	0.5142	0.029*	
H18B	0.8337	−0.1867	0.5386	0.029*	
C19	0.91156 (19)	−0.3754 (2)	0.53860 (5)	0.0245 (5)	
H19A	0.9968	−0.3496	0.5451	0.029*	
H19B	0.9190	−0.4472	0.5227	0.029*	
C20	0.84121 (19)	−0.4277 (2)	0.56855 (5)	0.0246 (5)	
H20A	0.8313	−0.3552	0.5842	0.030*	
H20B	0.7569	−0.4565	0.5620	0.030*	
C21	0.9087 (2)	−0.5439 (2)	0.58526 (5)	0.0260 (5)	
H21A	0.9129	−0.6187	0.5701	0.031*	
H21B	0.9951	−0.5172	0.5903	0.031*	
C22	0.8444 (2)	−0.5903 (2)	0.61656 (5)	0.0282 (5)	
H22A	0.8427	−0.5161	0.6319	0.034*	
H22B	0.7570	−0.6136	0.6116	0.034*	
C23	0.90728 (19)	−0.7093 (2)	0.63310 (5)	0.0266 (5)	
H23A	0.9130	−0.7823	0.6175	0.032*	
H23B	0.9932	−0.6846	0.6393	0.032*	
C24	0.8373 (2)	−0.7579 (2)	0.66318 (5)	0.0347 (6)	
H24A	0.7505	−0.7799	0.6572	0.042*	
H24B	0.8342	−0.6861	0.6792	0.042*	
C25	0.8993 (2)	−0.8805 (3)	0.67877 (6)	0.0446 (7)	
H25A	0.8492	−0.9095	0.6971	0.067*	
H25B	0.9833	−0.8577	0.6861	0.067*	
H25C	0.9043	−0.9513	0.6629	0.067*	
H1O1	1.046 (3)	0.654 (3)	0.1909 (7)	0.078 (10)*	

### Atomic displacement parameters (Å^2^)

**Table:**

	*U*^11^	*U*^22^	*U*^33^	*U*^12^	*U*^13^	*U*^23^
O1	0.0271 (8)	0.0216 (8)	0.0209 (7)	0.0019 (7)	0.0033 (6)	0.0032 (6)
O2	0.0224 (7)	0.0248 (8)	0.0213 (7)	0.0034 (7)	0.0014 (6)	0.0005 (6)
O3	0.0228 (7)	0.0231 (8)	0.0222 (7)	0.0004 (6)	0.0017 (6)	0.0052 (6)
C1	0.0207 (10)	0.0187 (11)	0.0245 (10)	0.0008 (9)	0.0005 (8)	−0.0032 (9)
C2	0.0230 (10)	0.0175 (11)	0.0255 (11)	0.0006 (9)	0.0002 (8)	0.0024 (9)
C3	0.0239 (10)	0.0159 (11)	0.0195 (10)	−0.0047 (9)	−0.0001 (8)	0.0004 (8)
C4	0.0239 (10)	0.0195 (11)	0.0237 (10)	0.0032 (9)	0.0025 (8)	−0.0016 (9)
C5	0.0235 (10)	0.0186 (11)	0.0236 (10)	0.0005 (9)	−0.0006 (8)	0.0010 (9)
C6	0.0212 (10)	0.0169 (11)	0.0198 (10)	−0.0021 (9)	−0.0010 (8)	−0.0018 (8)
C7	0.0226 (10)	0.0152 (10)	0.0190 (10)	−0.0001 (9)	−0.0018 (8)	−0.0048 (8)
C8	0.0203 (10)	0.0213 (11)	0.0203 (10)	0.0004 (9)	−0.0006 (8)	−0.0017 (8)
C9	0.0228 (10)	0.0205 (11)	0.0178 (10)	−0.0004 (9)	−0.0012 (8)	−0.0029 (8)
C10	0.0231 (10)	0.0208 (11)	0.0171 (9)	−0.0012 (9)	−0.0002 (8)	−0.0028 (8)
C11	0.0222 (10)	0.0246 (12)	0.0178 (10)	−0.0032 (9)	0.0008 (8)	0.0019 (9)
C12	0.0187 (10)	0.0265 (12)	0.0215 (10)	0.0000 (9)	0.0002 (8)	−0.0016 (9)
C13	0.0238 (10)	0.0187 (11)	0.0179 (9)	−0.0012 (9)	−0.0023 (8)	0.0002 (8)
C14	0.0223 (10)	0.0199 (11)	0.0216 (10)	−0.0029 (9)	0.0038 (8)	0.0011 (9)
C15	0.0213 (10)	0.0235 (12)	0.0221 (10)	0.0021 (9)	0.0005 (8)	−0.0017 (9)
C16	0.0249 (10)	0.0212 (11)	0.0207 (10)	−0.0017 (9)	0.0021 (8)	0.0030 (8)
C17	0.0224 (10)	0.0209 (11)	0.0245 (10)	−0.0031 (9)	−0.0022 (8)	−0.0002 (9)
C18	0.0260 (10)	0.0233 (12)	0.0230 (10)	−0.0006 (10)	−0.0015 (8)	0.0036 (9)
C19	0.0279 (11)	0.0223 (12)	0.0234 (10)	0.0014 (10)	0.0018 (9)	0.0011 (9)
C20	0.0249 (11)	0.0235 (12)	0.0255 (11)	−0.0018 (10)	−0.0006 (9)	0.0032 (9)
C21	0.0272 (11)	0.0249 (12)	0.0261 (11)	0.0006 (10)	0.0009 (9)	0.0037 (9)
C22	0.0261 (11)	0.0269 (12)	0.0315 (12)	0.0029 (10)	0.0007 (9)	0.0071 (10)
C23	0.0260 (11)	0.0270 (12)	0.0268 (11)	0.0001 (10)	0.0005 (9)	0.0046 (9)
C24	0.0357 (13)	0.0337 (14)	0.0346 (12)	0.0097 (12)	0.0066 (10)	0.0125 (10)
C25	0.0445 (15)	0.0485 (17)	0.0408 (14)	0.0177 (13)	0.0110 (12)	0.0201 (13)

### Geometric parameters (Å, °)

**Table:**

O1—C3	1.354 (2)	C16—C17	1.508 (3)
O1—H1O1	0.94 (3)	C16—H16A	0.97
O2—C7	1.239 (2)	C16—H16B	0.97
O3—C13	1.373 (2)	C17—C18	1.520 (3)
O3—C16	1.443 (2)	C17—H17A	0.97
C1—C2	1.382 (3)	C17—H17B	0.97
C1—C6	1.400 (3)	C18—C19	1.522 (3)
C1—H1	0.93	C18—H18A	0.97
C2—C3	1.395 (3)	C18—H18B	0.97
C2—H2	0.93	C19—C20	1.522 (3)
C3—C4	1.392 (3)	C19—H19A	0.97
C4—C5	1.385 (3)	C19—H19B	0.97
C4—H4	0.93	C20—C21	1.522 (3)
C5—C6	1.399 (3)	C20—H20A	0.97
C5—H5	0.93	C20—H20B	0.97
C6—C7	1.479 (3)	C21—C22	1.518 (3)
C7—C8	1.477 (3)	C21—H21A	0.97
C8—C9	1.340 (3)	C21—H21B	0.97
C8—H8	0.93	C22—C23	1.518 (3)
C9—C10	1.461 (3)	C22—H22A	0.97
C9—H9	0.93	C22—H22B	0.97
C10—C15	1.396 (3)	C23—C24	1.513 (3)
C10—C11	1.403 (3)	C23—H23A	0.97
C11—C12	1.376 (3)	C23—H23B	0.97
C11—H11	0.93	C24—C25	1.526 (3)
C12—C13	1.397 (3)	C24—H24A	0.97
C12—H12	0.93	C24—H24B	0.97
C13—C14	1.384 (3)	C25—H25A	0.96
C14—C15	1.393 (3)	C25—H25B	0.96
C14—H14	0.93	C25—H25C	0.96
C15—H15	0.93		
			
C3—O1—H1O1	115.1 (18)	C16—C17—H17A	109.2
C13—O3—C16	117.84 (15)	C18—C17—H17A	109.2
C2—C1—C6	121.44 (19)	C16—C17—H17B	109.2
C2—C1—H1	119.3	C18—C17—H17B	109.2
C6—C1—H1	119.3	H17A—C17—H17B	107.9
C1—C2—C3	119.77 (19)	C17—C18—C19	113.01 (17)
C1—C2—H2	120.1	C17—C18—H18A	109.0
C3—C2—H2	120.1	C19—C18—H18A	109.0
O1—C3—C4	122.77 (18)	C17—C18—H18B	109.0
O1—C3—C2	117.63 (18)	C19—C18—H18B	109.0
C4—C3—C2	119.60 (18)	H18A—C18—H18B	107.8
C5—C4—C3	120.12 (19)	C18—C19—C20	113.81 (17)
C5—C4—H4	119.9	C18—C19—H19A	108.8
C3—C4—H4	119.9	C20—C19—H19A	108.8
C4—C5—C6	121.03 (19)	C18—C19—H19B	108.8
C4—C5—H5	119.5	C20—C19—H19B	108.8
C6—C5—H5	119.5	H19A—C19—H19B	107.7
C5—C6—C1	117.92 (18)	C21—C20—C19	113.30 (17)
C5—C6—C7	122.43 (18)	C21—C20—H20A	108.9
C1—C6—C7	119.38 (17)	C19—C20—H20A	108.9
O2—C7—C8	119.34 (17)	C21—C20—H20B	108.9
O2—C7—C6	120.14 (18)	C19—C20—H20B	108.9
C8—C7—C6	120.48 (17)	H20A—C20—H20B	107.7
C9—C8—C7	120.40 (18)	C22—C21—C20	113.74 (17)
C9—C8—H8	119.8	C22—C21—H21A	108.8
C7—C8—H8	119.8	C20—C21—H21A	108.8
C8—C9—C10	128.13 (19)	C22—C21—H21B	108.8
C8—C9—H9	115.9	C20—C21—H21B	108.8
C10—C9—H9	115.9	H21A—C21—H21B	107.7
C15—C10—C11	117.26 (18)	C23—C22—C21	114.78 (17)
C15—C10—C9	119.05 (18)	C23—C22—H22A	108.6
C11—C10—C9	123.63 (18)	C21—C22—H22A	108.6
C12—C11—C10	121.05 (18)	C23—C22—H22B	108.6
C12—C11—H11	119.5	C21—C22—H22B	108.6
C10—C11—H11	119.5	H22A—C22—H22B	107.5
C11—C12—C13	120.53 (19)	C24—C23—C22	113.59 (18)
C11—C12—H12	119.7	C24—C23—H23A	108.8
C13—C12—H12	119.7	C22—C23—H23A	108.8
O3—C13—C14	124.86 (17)	C24—C23—H23B	108.8
O3—C13—C12	115.28 (17)	C22—C23—H23B	108.8
C14—C13—C12	119.86 (18)	H23A—C23—H23B	107.7
C13—C14—C15	118.92 (18)	C23—C24—C25	112.84 (19)
C13—C14—H14	120.5	C23—C24—H24A	109.0
C15—C14—H14	120.5	C25—C24—H24A	109.0
C14—C15—C10	122.36 (19)	C23—C24—H24B	109.0
C14—C15—H15	118.8	C25—C24—H24B	109.0
C10—C15—H15	118.8	H24A—C24—H24B	107.8
O3—C16—C17	107.35 (15)	C24—C25—H25A	109.5
O3—C16—H16A	110.2	C24—C25—H25B	109.5
C17—C16—H16A	110.2	H25A—C25—H25B	109.5
O3—C16—H16B	110.2	C24—C25—H25C	109.5
C17—C16—H16B	110.2	H25A—C25—H25C	109.5
H16A—C16—H16B	108.5	H25B—C25—H25C	109.5
C16—C17—C18	111.91 (17)		
			
C6—C1—C2—C3	0.5 (3)	C9—C10—C11—C12	−177.86 (19)
C1—C2—C3—O1	176.56 (18)	C10—C11—C12—C13	−0.6 (3)
C1—C2—C3—C4	−3.1 (3)	C16—O3—C13—C14	−1.2 (3)
O1—C3—C4—C5	−176.75 (18)	C16—O3—C13—C12	179.30 (17)
C2—C3—C4—C5	2.9 (3)	C11—C12—C13—O3	−179.39 (17)
C3—C4—C5—C6	−0.1 (3)	C11—C12—C13—C14	1.1 (3)
C4—C5—C6—C1	−2.5 (3)	O3—C13—C14—C15	−179.93 (18)
C4—C5—C6—C7	171.60 (18)	C12—C13—C14—C15	−0.5 (3)
C2—C1—C6—C5	2.3 (3)	C13—C14—C15—C10	−0.7 (3)
C2—C1—C6—C7	−171.99 (18)	C11—C10—C15—C14	1.2 (3)
C5—C6—C7—O2	−160.79 (19)	C9—C10—C15—C14	178.62 (19)
C1—C6—C7—O2	13.2 (3)	C13—O3—C16—C17	175.86 (16)
C5—C6—C7—C8	17.0 (3)	O3—C16—C17—C18	178.90 (16)
C1—C6—C7—C8	−168.94 (18)	C16—C17—C18—C19	177.92 (17)
O2—C7—C8—C9	5.4 (3)	C17—C18—C19—C20	175.44 (17)
C6—C7—C8—C9	−172.41 (18)	C18—C19—C20—C21	−178.11 (18)
C7—C8—C9—C10	177.52 (18)	C19—C20—C21—C22	175.79 (18)
C8—C9—C10—C15	−179.9 (2)	C20—C21—C22—C23	177.99 (18)
C8—C9—C10—C11	−2.7 (3)	C21—C22—C23—C24	−176.90 (19)
C15—C10—C11—C12	−0.6 (3)	C22—C23—C24—C25	178.0 (2)

### Hydrogen-bond geometry (Å, °)

**Table:**

*D*—H···*A*	*D*—H	H···*A*	*D*···*A*	*D*—H···*A*
O1—H1O1···O2^i^	0.95 (3)	1.71 (3)	2.655 (2)	177 (3)
C5—H5···O1^ii^	0.93	2.48	3.340 (2)	155

Symmetry codes: (i) *x*+1/2, *y*, −*z*+1/2; (ii) −*x*+2, *y*−1/2, −*z*+1/2.
